# A commentary on ‘“Dumpling suture method” versus traditional suture method of protective loop ileostomy in laparoscopic anterior rectal resection with specimen extraction through stoma incision: a retrospective comparative cohort study’

**DOI:** 10.1097/JS9.0000000000001503

**Published:** 2024-05-03

**Authors:** Qianqian Huang, Qiang Hu

**Affiliations:** Department of General Surgery, Tongde Hospital of Zhejiang Province, Hangzhou, People’s Republic of China

*Dear Editor,*

We have read with interest the article published by Gu *et al*.^1^. This study provides an innovative surgical technique, which is safe and effective in reducing the incidence of permanent skin infections and stone composites. We believe that this is a very meaningful study and it will play an important guiding role in clinical practice. Although we believe it is a very interesting topic, we would like to offer the following points for your consideration.

First, in this article, the nutritional evaluations of the two groups were not provided, such as the nutrition risk screening 2002 (NRS-2002) score. Tumor progression is often accompanied by uncontrolled consumption of nutrients, leading to malnutrition in patients with malignant tumors. Due to the unique location of the lesion, rectal cancer patients often suffer from obstacles in nutrient uptake, digestion, and absorption. At the same time, rectal cancer cells also release some cytokines, such as tumor necrosis factor alpha (TNF-α), which further aggravates the body’s metabolic process. Compared to other non-gastrointestinal tumor patients, malnutrition is more common in rectal cancer patients, with an incidence rate reported in the literature ranging from 29.1% to 80.4%^2^. Nutritional status is an important factor affecting postoperative recovery of the body. Malnutrition can affect the healing of surgical incisions, reduce the body’s ability to resist infections, increase postoperative complications, and further lead to prolonged hospitalization. At the same time, it can also affect the quality of life and clinical outcomes of patients. For patients with nutritional risk and malnutrition, timely nutritional intervention should be carried out. They have important clinical significance in promoting early recovery of patients, reducing postoperative complications, and improving long-term prognosis.

Second, in this article, the number of lymph node dissections was not given for both groups. We all know that a larger number of lymph node dissections can improve the accuracy of tumor staging, resulting in a more accurate prognosis evaluation, and more thorough lymph node dissection may lead to a better prognosis^3^. However, more thorough lymph node dissection may indeed make blood vessels too naked and fragile, making them prone to serious complications such as intra-abdominal bleeding. These complications may lead to prolonged hospital stays.

Third, in this article, the hemoglobin values for both groups are not given. Oxygen enters the bloodstream and combines with hemoglobin to form oxygenated hemoglobin, which is then transported to various parts of the body. Oxygen is an essential substance during the healing process of the anastomotic site. If a patient experiences low hemoglobin levels, the amount of oxygen reaching the anastomotic site will inevitably be lower than that of patients with normal hemoglobin levels, which may lead to the occurrence of anastomotic leakage. A large retrospective study covering 524 patients confirmed that preoperative anemia is an independent risk factor for postoperative complications in laparoscopic colon cancer radical surgery^4^. An analysis of postoperative complications in 1876 patients undergoing radical surgery for colorectal cancer found that hemoglobin levels less than 110 g were an independent risk factor for postoperative intestinal complications^5^.

In conclusion, the above factors need to be considered in this study.

## Ethical approval

The type of this manuscript is correspondence, which is not applicable.

## Consent

The type of this manuscript is correspondence, which is not applicable.

## Sources of funding

This study was funded by the Science and Technology Planning Project of Zhejiang Province (No. 2017F30045), Science and Technology Planning Project of Traditional Chinese Medicine (No. 2018ZZ004), Gastrointestinal Surgery of Integrated traditional Chinese and Western Medicine (No. 2017-XK-A20), and Project of Zhejiang Provincial Department of Education (Y202351378).

## Author contribution

Q.Huang: methodology, writing, and review; Q.Hu: conceptualization, methodology, writing, original draft, preparation, review and editing, and supervision.

## Conflicts of interest disclosure

The authors declare that they have no conflicts of interest.

## Research registration unique identifying number (UIN)

The type of this manuscript is correspondence, which is not applicable.

## Guarantor

Qiang Hu.

## Data availability statement

This manuscript is a comment. Don’t need a data availability statement. However, all the data from the current study are publicly available.

## Provenance and peer review

My manuscript was not invited.
